# Compassionate use of cefiderocol in a complex case of extensively drug-resistant *Acinetobacter baumannii* fracture-related infection: a comprehensive approach and multidisciplinary management

**DOI:** 10.1007/s15010-024-02294-x

**Published:** 2024-05-15

**Authors:** Flavia Petrucci, Beatrice Perciballi, Marco Rivano Capparuccia, Giancarlo Iaiani, Federico Lo Torto, Diego Ribuffo, Stefano Gumina, Daniele De Meo

**Affiliations:** 1 Department of Public Health and Infectious Diseases, Sapienza University of Rome, 00185 Rome, Italy; 2 M.I.T.O. (Infections in Traumatology and Orthopedics Surgery) Study Group, Policlinico Umberto I Hospital, Viale del Policlinico 155, 00161 Rome, Italy; 3 Department of Anatomical, Forensic Medicine and Musculoskeletal System Sciences, Sapienza University of Rome, 00161 Rome, Italy; 4 Department of Internal Medicine, Endocrine-Metabolic Sciences and Infectious Diseases, Policlinico Umberto I University Hospital, 00161, Rome, Italy; 5 Plastic Surgery Unit, Department of General Surgery, Plastic Surgery, Orthopedics Policlinico Umberto I Hospital-Sapienza, University of Rome, Viale del Policlinico 155, 00161 Rome, Italy

**Keywords:** Antimicrobial resistance, Fracture related infection, Tibia fracture, *Acinetobacterbaumannii*, Cefiderocol, Multidisciplinary

## Abstract

**Purpose:**

Fracture-related infections (FRI) pose a difficult management problem, as they require numerous surgical interventions and extended antibiotic treatments, especially when a multidrug-resistant organism is involved, with a paucity of available literature that provides guidance.

**Results:**

A 42 year-old male presents an open diaphyseal tibia and fibula fracture, complicated by soft tissue necrosis and infections caused by extensively drug-resistant *Acinetobacter baumannii* (XDR-Ab). Initially treated with a damage control external fixator, the patient underwent multiple surgical procedures, including radical debridement, negative pressure wound therapy, external fixator revisions and reconstructive surgery using a latissimus dorsi free flap. The emergence of colistin resistance in the *Acinetobacter baumannii* strain led to the compassionate use of cefiderocol, finally achieving clinical cure.

**Conclusions:**

This case report is one of the firsts that highlights the potential efficacy of cefiderocol in treating challenging bone and joint infections sustained by XDR-Ab. The successful outcome also emphasizes the importance of a comprehensive, multidisciplinary approach in achieving favorable results in complex FRI.

## Introduction

Fracture-related infection (FRI) is a severe complication following bone injury that requires multiple operations or even amputation to save the patient’s life, causing a major problem for orthopedic surgeons as well as a serious social and economic burden [1]; in order to overcome this issues, multidisciplinary approaches are needed with orthopedic, infectious disease specialist and plastic surgeon combined and timely treatment. *Acinetobacter baumannii* (Ab) is a Gram-negative, aerobic, pleomorphic and non-motile bacillus, and it is an opportunistic pathogen primarily associated with hospital-acquired infections, representing a major clinical challenge due to its mechanisms of antimicrobial resistance [2, 3]. When a FRI is sustained by a germ of this kind, with few therapeutic options, poses an even greater challenge. Cefiderocol (FDC) is a new siderophore cephalosporin, authorized for infections caused by carbapenem-resistant gram-negative bacteria, and here we present the case of a fracture-related infection successfully treated with FDC requested under compassionate use as rescue therapy since the development of colistin resistance by *Acinetobacter baumannii*.

## Case presentation

**Fig.1:**
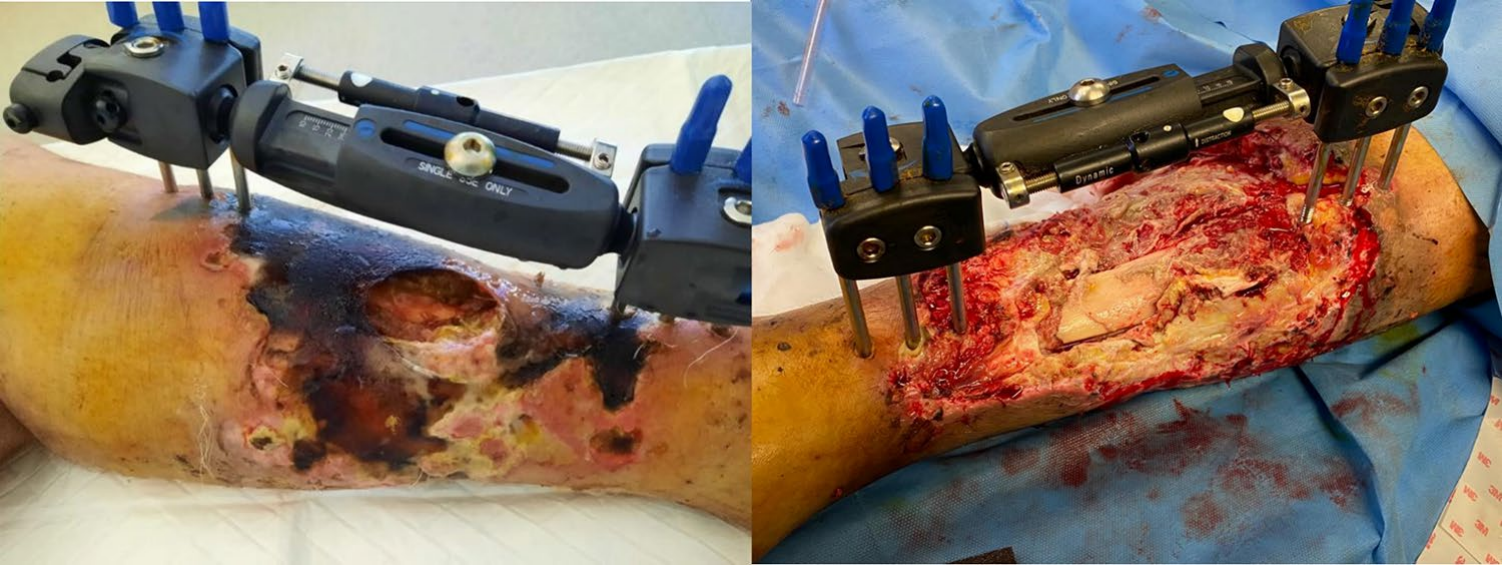
Skin condition with the first external fixator placed, before and after the debridement

**Fig.2:**
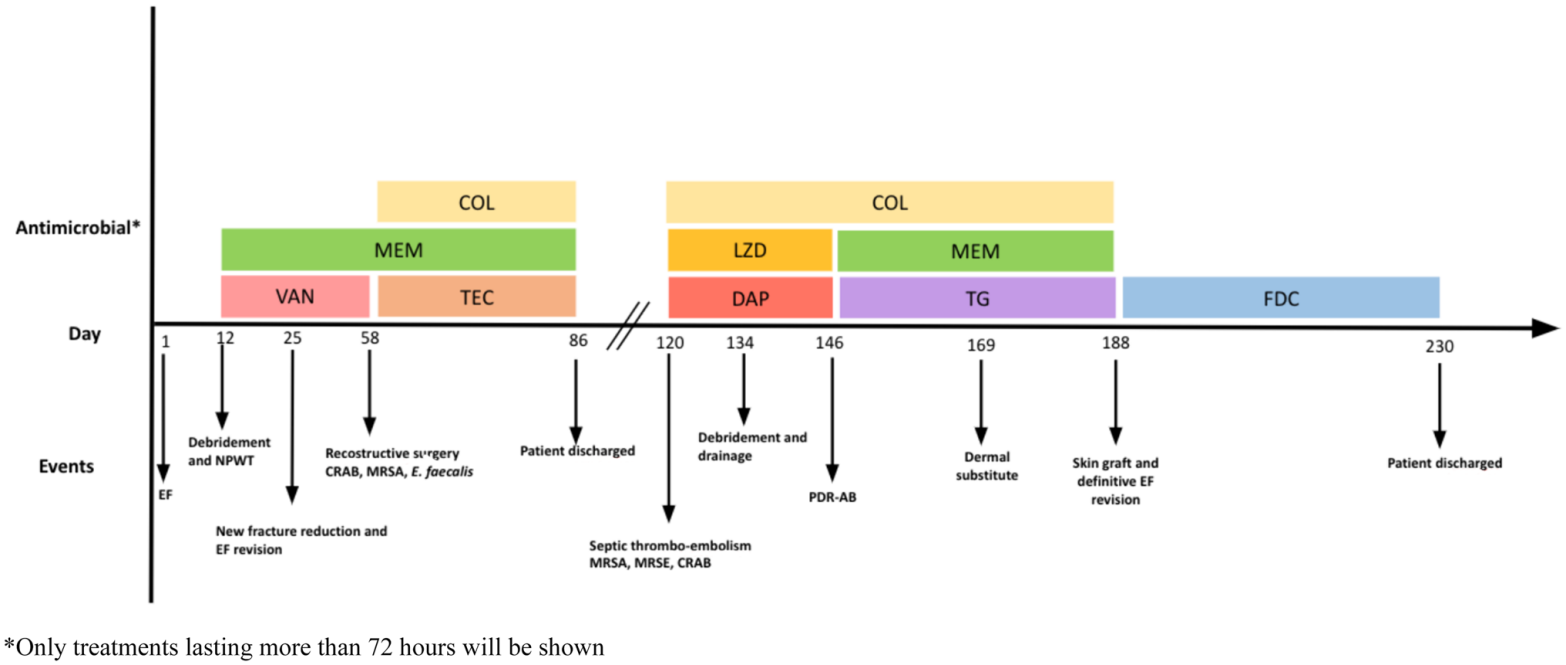
Main in-hospital events and antibiotics timeline. *COL* colistin, *MEM* meropenem, *VAN* vancomycin, TEC teicoplanin, *NPWT* negative pressure wound therapy, EF external fixator, CRAB carbapenem-resistant *Acinetobacter* *baumannii*, *MRSA* methicillin-resistant Staphylococcus aureus, *MRSE* methicillin-resistant Staphylococcus epidermidis, *PDR-AB* pandrug-resistant *Acinetobacter* *baumannii*, *LZD* linezolid, *DAP* daptomycin, TG tigecycline, *FDC* cefiderocol. *Only treatments lasting more than 72 h will be shown

A 42 year-old male patient with an open diaphyseal tibia and fibula fracture of the left leg (Gustilo 3b, AO 42A2,4F2B), resulting from a car accident, initially received treatment at a peripheral hospital with a damage control external fixator. His previous medical history was not relevant, except for a prior left sciatic nerve deficit due to chronic radiculopathy. Subsequently, he was transferred to our institution (Umberto I University Hospital, Rome) after 12 days, presenting with severe soft tissue necrosis. Soon after his arrival, he underwent radical debridement and the placement of negative pressure wound therapy (NPWT) (Fig.1). Empirical antibiotic treatment with meropenem (MEM) and vancomycin (VAN) was initiated (Fig. 2).

In the month following the initial surgery, the patient underwent serial debridement and NPWT renewal. Almost one month after trauma, a new fracture debridement and reduction with external fixation revision were performed, transitioning from a damage control construct to a monoplanar definitive one (Procallus—Orthofix).

Two months after trauma, the plastic surgery team conducted reconstructive surgery for soft tissue loss using a latissimus dorsi free flap. Intraoperative samples collected were positive for methicillin-resistant *Staphylococcus aureus* (MRSA), *Enterococcus faecalis* (susceptible to ampicillin), and carbapenem-resistant *Acinetobacter baumannii* (CRAB), susceptible only to colistin (MIC = 0.5 mcg/mL). Given the antibiotic options available at our hospital at that time, therapy with MEM, teicoplanin (TEC), and colistin (COL) was initiated. After four weeks of antibiotics, with observed improvement, the patient was discharged.

During the outpatient follow-up four months after the trauma, the patient exhibited flap swelling, decubitus on the fixator body, and modest leakage of proximal pins. Consequently, the external fixator was elevated, and empiric therapy with levofloxacin (LVX) and minocycline (MIN) was prescribed. However, after two days, the patient was readmitted to the peripheral hospital in a confused and febrile state, with severe dyspnea, and diagnosed with septic thrombo-embolism.

**Fig.3:**
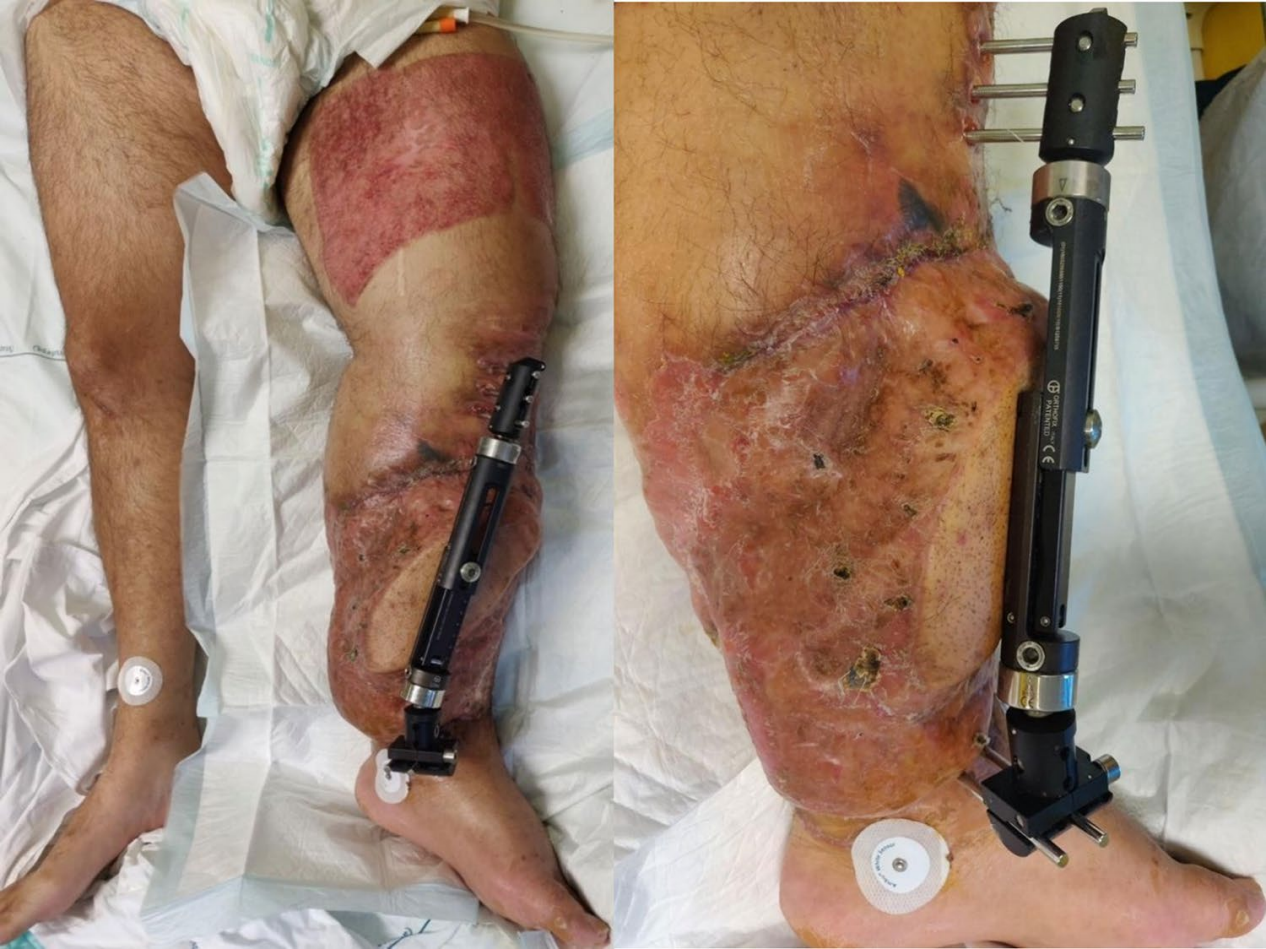
Left leg presentation after septic thrombus embolism episode

The patient initiated antimicrobial therapy with daptomycin (DAP), linezolid (LZD), and COL, as the blood cultures were positive for MRSA, and the skin swabs collected were positive for methicillin-resistant *Staphylococcus epidermidis* (MRSE) and CRAB. One week after admission, he was transferred to the Department of Infectious and Tropical Diseases of our hospital. Physical examination revealed edema in the left lower limb, purulent discharge from the proximal pins, and positive thermotactile response over the entire knee and leg region (Fig. 3).

Magnetic resonance imaging revealed corpuscular fluid collections at the anterolateral compartment of the left leg. Subsequently, source control surgery was performed, involving debridement, drainage of the collections, NPWT placement, and progressive skin closure using a “shoelace technique”. Simultaneously, MRSA and CRAB were isolated from biopsy. Two weeks after source control surgery, following the isolation of an Ab strain resistant to colistin (MIC > 4 mcg/mL) from biopsies during NPWT renewal, LZD and DAP were discontinued, and therapy continued with COL, MEM, and tigecycline (TG).

**Fig. 4:**
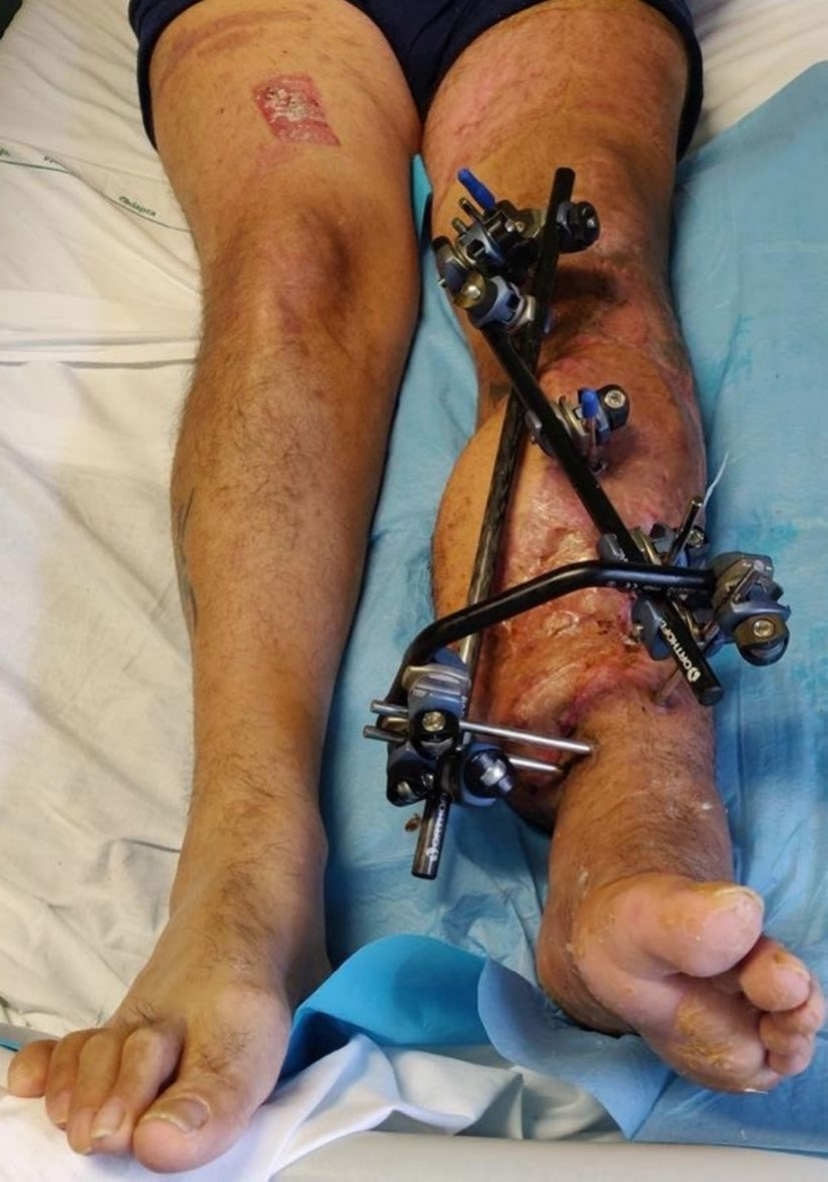
Last combined ortho-plastic surgery with multiplanar external fixator

The patient’s soft tissue condition progressively improved until five weeks after source control surgery when NPWT was removed, and a dermal substitute was placed until the final combined ortho-plastic surgery eight weeks after source control surgery (approximately seven months after the initial trauma). During this surgery, a partial-thickness epidermal graft was placed, and the external fixator was stabilized by converting it to a multiplanar system (Galaxy System—Orthofix) (Fig. 4). No antibiotic delivery systems were employed, in line with our hospital’s policy, which suggests their utilization in the presence of internal fixation devices [4] or in cases involving substantial joint or endosteal collections.

On the same day, since the susceptibility of the isolated strain to cefiderocol (FDC) was determined in the meantime, the patient discontinued the previous antibiotic therapy and initiated FDC on a compassionate basis as rescue therapy. He received 2 g every 8 h via a 3 h infusion for six weeks without experiencing side effects.

**Fig.5:**
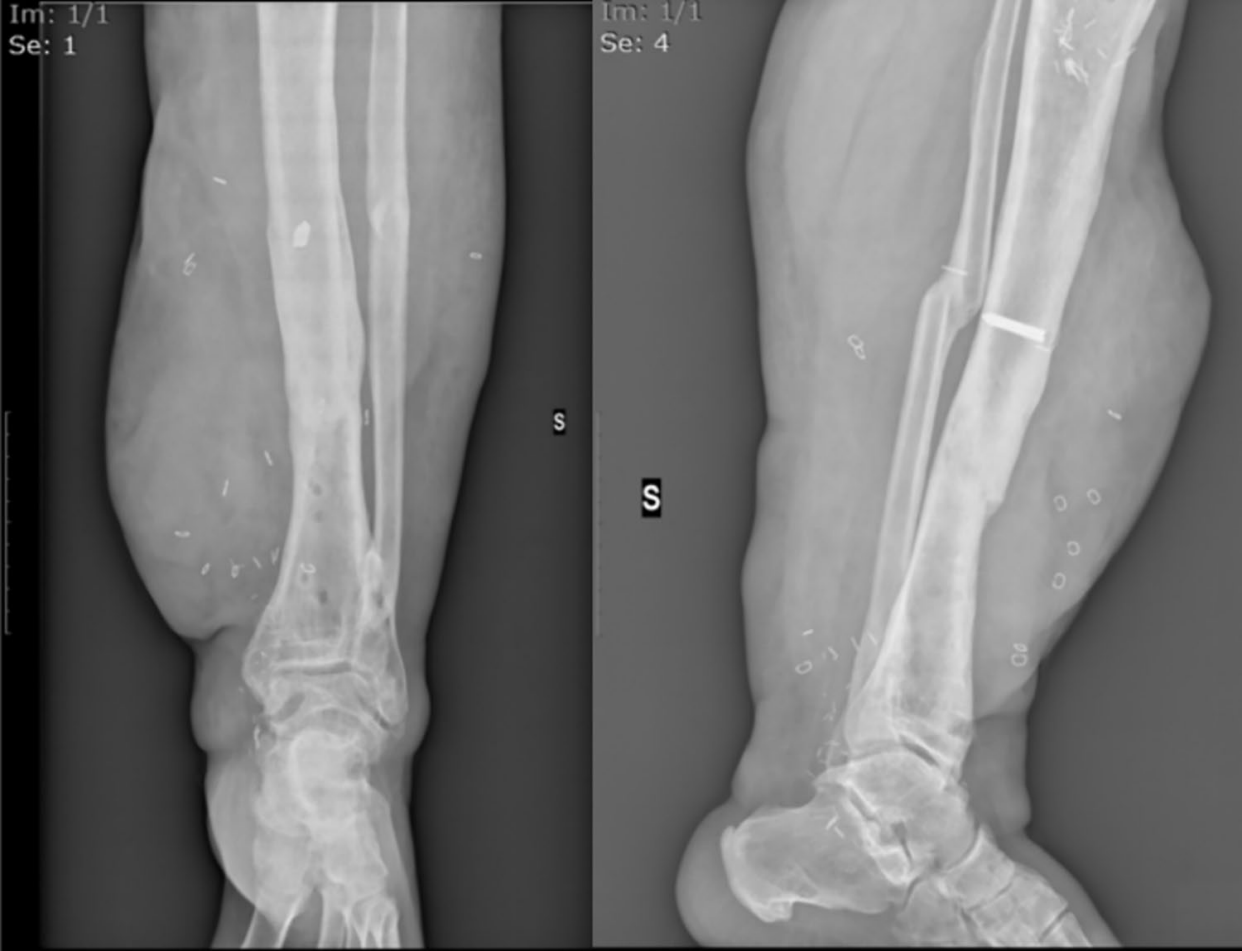
Radiographs post external fixator removal with evident bone consolidation

After six weeks, he was discharged from the Department of Infectious and Tropical Diseases, continuing his treatment course with clinical and radiographic follow-ups at our bone infection outpatient clinic at Umberto I University Hospital on a monthly basis. Serial X-ray examinations showed very slow progressive bone callus formation. Finally, two years after the initial trauma, the external fixator was removed (Fig. 5). Concurrently, the skin condition continued to improve, leading to flap debulking surgery three years after the flap surgery.

At the last available checkup conducted three years after patient discharge, the patient reported having returned to his daily activities, walking independently without the need for aids, pain-free, and with a healing skin condition.

## Discussion

When a FRI occurs, occasionally, soft tissue defects are of significant size, necessitating multiple surgical debridements and the provisional application of NPWT until the feasibility of definitive soft tissue coverage. Throughout this hospitalization period, pathogens known to cause hospital-acquired infections can become prominent players in FRI development. In this context, this case report underscores several critical antimicrobial aspects in FRI management. One of the primary challenges encountered was the presence of multiple antibiotic-resistant bacteria, including CRAB, MRSA and MRSE, necessitating careful selection of antimicrobial agents guided by culture and susceptibility testing.

Ab is often associated with healthcare settings, especially Intensive Care Units and surgical wards given its ability to persist on surfaces and to aerosolize. Therefore, patients who have undergone invasive procedures, surgeries, or prolonged hospitalizations are at higher risk of acquiring Ab infections, including osteomyelitis. Studies on American soldiers have shown an increase in this type of infection in war wounds, with a progressive shift towards nosocomial acquisition [5]. More recent Chinese studies [6, 7] have shown a progressive increase in FRI sustained by Gram-negative multidrug resistant organism (MDROs). Unfortunately consistent data about bone infections sustained by Gram-negative MDROs in Europe are lacking, but according to the latest ECDC data in Italy, Ab isolates are carbapenem-resistant in 84% of cases [8]. Hence, we can easily expect an increasing number of FRI sustained by Gram-negative MDROs, especially CRAB.

Until now colistin has always been considered the backbone therapy for CRAB, and the available guidelines, both recent and past, suggest the central role of this drug, underlining the complexity of managing such infections [9, 10]. The eradication of MDROs from bone tissue presents a significant management challenge, given the limited availability of active antimicrobials, the difficulty in penetrating bone tissue and the potential drug-related toxicity. Only clinical case reports discuss the management of bone infections caused by MDROs [11–13]: Siebenbürger et al. [14], describe the microbiological eradication of Ab and *E. cloacae* with a regimen of both intravenous and local colistin, and Pasticci et al. [15] reports the tolerability of a long regimen with colistin while treating a polymicrobial prosthetic joint infection.

Furthermore, the development of colistin resistance by Ab, besides being a negative prognostic factor, highlights the challenges associated with managing such infections. Consequently, after weeks of colistin based therapy, we requested cefiderocol on a compassionate basis as a rescue therapy for our patient.

Cefiderocol is a new siderophore cephalosporin approved for the treatment of carbapenem-resistant gram-negative bacterial infections [16]. Even if CREDIBLE-CR [17] study has raised doubts about its effectiveness in treating extensively resistant (XDR)-Ab infections, real life experiences of complex infections successfully treated with FDC are steadily rising. We found five other similar cases (table 1): among these, Dagher at al. [19], describe XDR-Ab osteomyelitis treated with FDC for 109 days, achieving both clinical and microbiological cure. It is interesting to note that all cases were supported by colistin-sensitive Ab, unlike ours, which had developed resistance.

**Table 1:** Complicated infections caused by extensively drug resistant *Acinetobacter baumannii* treated with cefiderocol

Case	Diagnosis	Dosage of FDC	Duration of FDC therapy, days	Clinical and microbiological outcome
Oliva et al. [18], 55 y, female	XDR-Ab spondylodiscitis	2 g q8h	21	Clinical cure at 9 month follow-up; clearance of XDR- Ab from the bone
Dagher et al. [19], 57 y, male	Polymicrobial osteomyelitis (XDR-Ab, *Enterococcus* *faecalis*, *Corynebacterium* *striatum*)	2 g q8h	109	Clinical and microbiological cure
Zingg et al. [20], 29 y, male	Acute polymicrobial osteomyelitis (VIM- producer *P*. *aeruginosa*, OXA-23 *A*. *baumannii*, KPC producer *Enterobacter* *cloacae*)	Not specified	14	Clinical and microbiological cure
Zingg et al. [20], 64 y, male	Early postoperative implant-associated infection of the spine (OXA-40 and NDM- producer *A*. *baumannii*)	1.5 g q8h (after adjustment for renal clearance)	54	Clinical cure
Mabayoje et al. [21], 66 y, female	XDR-Ab prosthetic joint infection	1.5 g q8h (after adjustment for renal clearance)	25	Clinical cure

*XDR-Ab* extensively drug resistant *Acinetobacter baumannii*, *FDC* cefiderocol

Alamarat et al., [22] also describe long-term (14 weeks) compassionate use of FDC to treat chronic osteomyelitis caused by XDR *Pseudomonas aeruginosa* and ESBL *Klebsiella* *pneumoniae* in a pediatric patient, resulting in apparent cure and avoided amputation. All these cases, along with the one described here, seem to support the role of FDC in treating complex XDR-Ab infections, especially when biofilm is involved. This is probably due to its action on the bacterial iron-transport system because iron is crucial for biofilm formation, and siderophore production is upregulated in biofilms [23].

One of the key points highlighted in this case is the complexity and challenges associated with managing open fractures, especially those complicated by soft tissue necrosis and subsequent infections. The initial presentation of soft tissue necrosis necessitated aggressive multiple debridements and NPWT placement to promote wound healing and control of the infection. The multidisciplinary approach involving plastic surgery, infectious diseases, and orthopedic surgery was crucial in managing the various aspects of the patient's condition, including wound care, infection control, and surgical interventions. Source control surgeries, including debridement and drainage of fluid collections, were essential in managing persistent infections and preventing further complications.

The patient's return to normal activities with a healing skin condition signifies a favorable prognosis and underscores the importance of comprehensive management strategies in achieving successful outcomes in complex cases of FRI.

## Conclusions

This case report illustrates the challenges and complexities involved in the management of FRI especially when a multidrug-resistant organism is involved. A multidisciplinary approach incorporating surgical intervention, antibiotic therapy, wound care, and long-term follow-up is essential for achieving optimal outcomes in such cases. The good outcome achieved, although we cannot attribute it to FDC only, supports, in line with the literature, the use of this antibiotic in managing these very complex infections.

## Data Availability

No datasets were generated or analysed during the current study.
