# Correction: Compassionate use of cefiderocol in a complex case of extensively drug-resistant Acinetobacter baumannii fracture-related infection: a comprehensive approach and multidisciplinary management

**DOI:** 10.1007/s15010-024-02306-w

**Published:** 2024-06-07

**Authors:** Flavia Petrucci, Beatrice Perciballi, Marco Rivano Capparuccia, Giancarlo Iaiani, Federico Lo Torto, Diego Ribuffo, Stefano Gumina, Daniele De Meo

**Affiliations:** 1 Department of Public Health and Infectious Diseases, Sapienza University of Rome, 00185 Rome, Italy; 2 M.I.T.O. (Infections in Traumatology and Orthopedics Surgery) Study Group, Policlinico Umberto I Hospital, Viale del Policlinico 155, 00161 Rome, Italy; 3 Department of Anatomical, Forensic Medicine and Musculoskeletal System Sciences, Sapienza University of Rome, 00161 Rome, Italy; 4 Department of Internal Medicine, Endocrine-Metabolic Sciences and Infectious Diseases, Policlinico Umberto I University Hospital, 00161 Rome, Italy; 5 Plastic Surgery Unit, Department of General Surgery, Plastic Surgery, Orthopedics Policlinico Umberto I Hospital—Sapienza, University of Rome, Viale del Policlinico 155, 00161 Rome, Italy

**Correction: Infection**

10.1007/s15010-024-02294-x

In this article first name and last name of the authors were interchanged.

Correct is:

Flavia, Petrucci^1,2^ · Beatrice, Perciballi^2,3^ · Marco, Rivano Capparuccia^2,4^ · Giancarlo, Iaiani^2,4^ · Federico, Lo Torto^2,5^ · Diego, Ribuffo^2,5^ · Stefano, Gumina^3^ · Daniele, De Meo^2,3^

The Original Article has been corrected.

